# How medical students’ early workplace experience could benefit the NHS

**DOI:** 10.1186/s12909-021-02768-8

**Published:** 2021-06-07

**Authors:** George Chukwuemeka Oyekwe, Muhammed Aizaz us Salam, Sami Ahmad Ghani, Bilal Iyad Abedalaziz Alriyahi

**Affiliations:** St George’s University of London, Cranmer Terrace, Tooting, London, SW17 0RE UK

**Keywords:** Early workplace experience, National Health Service, Communication, Medical student, Professional identity, Patient experience

## Abstract

As senior medical students who have had the privilege of undergoing early year clinical workplace experience, we believe that this opportunity medical students have through experiencing the ‘frontline’ could be utilised advantageously by the National Health Service (NHS). A system under pressure with depleting resources seemingly persists to be a constant theme that surrounds the NHS. Due to such issues, improvements in all sectors are sought, including costs, patient experience and communication between healthcare professionals. Through this article we highlight and analyse how medical students’ early workplace experience, as presented by Leedham-Green et al., could benefit the NHS through tackling some of the challenges mentioned prior.

## Background

We thank Leedham-Green et al. for their article, ‘Intra- and interprofessional practices through fresh eyes: a qualitative analysis of medical students’ early workplace experiences.’ The article aptly recognises a variety of collaborative practices within the healthcare environment and possible difficulties that can be encountered as a result of particular elements, such as the lack of communication between healthcare professionals [1]. By using the ‘hidden curricula’ to address what medical students are understanding about interprofessional dynamics, potential areas for quality improvement within the NHS can be identified. The NHS is facing seemingly ever-more strain with doctors being burdened with surging task-loads, resulting in time constraints, understaffing and excessive workload. These factors, as analysed by Leedham-Green et al., resulted in communication issues in the healthcare setting, as well as rising difficulties for newly qualified junior doctors to cope with their position. In this article we explore these key themes identified by Leedham-Green et al. We also reflect on how the perspective of medical students provides an alternative way to address these problems encountered within the NHS, and how undertaking early workplace experience allows medical students to develop their professional identities through patient care and eases the transition to becoming future competent doctors.

## Main text

Expectations medical students have upon the completion of their degree include being a competent doctor who can tackle any task they are handed, within their remit. However, with the NHS facing increasing demand and strain, it can be difficult to envision how the NHS can persevere. Therefore, we contemplated on what can be achieved for the betterment of the NHS. As senior medical students who have had the opportunity to acquire clinical experience in earlier years, we believe that such knowledge gained in those earlier years has had a pivotal role in our growth as future practitioners, hence positively contributing to the future of the NHS.

### The issue of communication

The majority of medical schools now implement an integrated programme through which early patient contact is encouraged, with only a few medical schools opting for more traditional curricula [2]. Medical student exposure to early-year clinical experience has enabled Leedham-Green et al. to exemplify routes whereby breakdown in communications and dynamics between healthcare professionals can occur; whether it be due to factors such as increasing workload, time constraints or understaffing.

Inadequate communication could negatively impact the quality of care that the patient receives [1]. Communication is an essential skill for doctors to possess. When newly qualified, they are assumed to be able to interact effectively with patients as well as colleagues, according to the General Medical Council (GMC) [3]. Leedham-Green et al. also provide instances of effective communication, despite obstacles that can be encountered in the NHS. With the ability to reflect on both adequate and dissatisfactory examples of communication in a healthcare setting, medical students are able to formulate methods based on how they themselves should portray themselves in future practice.

This experience gained in the early years of medical school could serve to benefit the NHS, especially with the implementation of the ‘NHS long term plan’; which aims to provide a “more joined up and coordinated care” [4]. In addition, the plan emphasises the significance of constructing genuine partnerships between professionals and patients, such that patients are able to manage their own health better [5]. This could be vital for chronic conditions such as diabetes, whereby defective communication between doctors and patients is often the crux of the issues that lead to non-compliance with medications and advised lifestyle changes [6]. The estimated number of people diagnosed with diabetes in the UK is 3.5 million with a further 549,000 people having undiagnosed diabetes. Moreover, the treatment of diabetes accounts for around 10% of the NHS’ yearly budget which equates to £9 billion [7]. Better informed patients regarding the management of their condition will reduce the likelihood of potential complications – further freeing up resources for other NHS demands.

With the implementation of the ‘NHS long term plan’ being a major reinforcement for the NHS, we believe it is paramount that medical students are not only apprised of these plans, but are also able to witness the execution of such reinforcements in their early year clinical experience, to apply this knowledge in their future practice.

### Developing a professional identity and its effects on patient experience

When it comes to medical students in their clinical placements, Leedham-Green et al. alludes to the importance of evaluating collaborative practices between healthcare professionals, as it is evident that the professional identities of medical students can be shaped by encounters in the clinical workplace [1]. A study by Weaver et al. explored which elements contribute to the formation of professional identity in medical students. In particular, professional inclusivity was found to be a contributing factor which medical students felt they experienced in clinical placements, when they were treated as future medical professionals by doctors and patients [8]. The GMC expects newly qualified doctors to ensure that the fundamental needs of patients are addressed, through demonstrating compassionate professional behaviour [3]. We believe that shaping the professional identity of medical students, through early patient contact, is favourable for both future doctors and patients. This is evident from a study conducted by Thistlethwaite et al., which investigated the views of patients regarding early student-patient interactions. It was apparent that a majority of patients valued the opportunity to aid medical students, as they felt that information passed on will help patients in the future. Furthermore, some patients found themselves satisfied by interacting with medical students, as they found it therapeutic to talk to new people about their condition [9].

Doctors encounter patients at some of the most vulnerable periods of their lives, with each interaction potentially leaving a longstanding reflection of the NHS embedded in their minds. With patient-centred care being a doctrine that the NHS upholds, it is essential that a high standard of professionalism is maintained by healthcare professionals in order to accomplish this. Through clinical settings in early years of medical school, students have the chance to develop their professional identity as future representatives of the NHS, as they can truly serve the needs of patients to an appropriate standard, and provide them with the care they need.

### Transitioning from a medical student to a junior doctor

‘Criticism of suboptimal interpersonal interactions’ is another key theme highlighted within Leedham-Green et al.’s article, with instances of lack of respect or support for junior members of staff [1]. Medical students viewing deplorable treatment of junior doctors, at such an early stage in their learning, could explain why some find the transition from a medical student to a junior doctor to be stressful. According to a report produced by the Royal College of Physicians (RCP) on the perspective of junior doctors working in the NHS, it is clearly evident that working in the NHS can be intense, challenging, however also rewarding. In addition, “four in five junior doctors regularly experience excessive stress because of their job” [10]. Therefore, it is crucial that strategies in reducing this stress are developed. Interestingly, a study conducted by Brennan et al. on the experience of junior doctors in their first year of clinical practice identified that most of the participants found the transition from being medical students to junior doctors to be taxing [11]. This was due to factors such as dealing with new responsibility, managing uncertainty and working in multi-disciplinary teams. On the other hand, some felt that the level of experience that they gained in their undergraduate years reduced the stress of transition [11]. Reflection of intra- and interprofessional practices by medical students, as expressed by Leedham-Green et al., provides an opportunity to identify areas for systematic improvement not only for staff wellbeing, but to also relieve potential stress of future doctors. Enabling medical students to gain experience from early patient contact, and integrating them as part of the healthcare team, allows them to transition into becoming junior doctors smoothly and potentially reduces future tension, which would ultimately assist the NHS.

The integration of students in the healthcare setting further allows them to develop their leadership qualities and helps them become ‘change agents’ to the future of the NHS. This incorporation would allow them to develop their critical reasoning and achieve the core competencies for effective teamwork in health systems, and this would only be achieved through the “promotion of interprofessional and transprofessional education that breaks down professional silos while enhancing collaborative and non-hierarchical relationships in effective teams.” [12] Through allowing students to participate in patient care, under the guidance of seniors, and reflect on various aspects of patient care, including the ethics of decision making, this would not only benefit the students in transitioning to junior doctors, but also the healthcare team through allowing the students’ perspectives to support patients’ management.

Early exposure to the clinical setting enables students to compare theoretical practice to actual practice, through performing activities such as attending ward rounds [1]. Applying knowledge gained from lecture-based settings not only reinforces learning, but also allows the potential comfort of the clinical environment to be found. As senior medical students with early year clinical experience, we perceive that this familiarity with the clinical setting, as well as interprofessional interaction, is essential for the development of competent doctors.

## Conclusion

The journey students take through medical school is filled with trials and tribulations that inevitably shape them as future doctors. With the NHS constantly trying to improve and adapt, it is important to remember the people who will form the continuing future of the NHS, and to incorporate them as part of this change. We agree with the conclusion established by Leedham-Green et al. that reflection of students’ experiences of intra- and interprofessional practice provides a chance for systems thinking and service analysis. By exploring the implications of clinical workplace experience gained in the early years of medical school, we have found promising potential benefits to the NHS. Such benefits include improved communication techniques which can be applied in the healthcare setting, patient experience and the potential to ease transition of medical students to junior doctors. In our opinion, this synergism between medical student’s early workplace experience and the NHS requires more research to be conducted, to inspire a positive change for the upcoming generations of healthcare professionals to form the NHS of tomorrow.

## Data Availability

Not applicable.

## Abbreviations

NHS: National Health Service
GMC: General Medical Council
RCP: Royal College of Physicians
MDT: Multi-disciplinary Team
